# The Impact of Socio-Demographic and Religious Factors upon Sexual Behavior among Ugandan University Students

**DOI:** 10.1371/journal.pone.0023670

**Published:** 2011-08-24

**Authors:** Anette Agardh, Gilbert Tumwine, Per-Olof Östergren

**Affiliations:** 1 Department of Clinical Sciences, Social Medicine and Global Health, Lund University, Malmö, Sweden; 2 Department of Obstetrics and Gynaecology, St. Francis Hospital Nsambya, Kampala, Uganda; University of Liverpool, United Kingdom

## Abstract

**Introduction:**

More knowledge is needed about structural factors in society that affect risky sexual behaviors. Educational institutions such as universities provide an opportune arena for interventions among young people. The aim of this study was to investigate the relationship between sociodemographic and religious factors and their impact on sexual behavior among university students in Uganda.

**Methods:**

In 2005, 980 university students (response rate 80%) were assessed by a self-administered questionnaire. Validated instruments were used to assess socio-demographic and religious factors and sexual behavior. Logistic regression analyses were applied.

**Results:**

Our findings indicated that 37% of the male and 49% of the female students had not previously had sex. Of those with sexual experience, 46% of the males and 23% of the females had had three or more sexual partners, and 32% of the males and 38% of the females did not consistently use condoms. For those who rated religion as less important in their family, the probability of early sexual activity and having had a high number of lifetime partners increased by a statistically significant amount (OR = 1.7; 95% CI: 1.2–2.4 and OR = 1.6; 95% CI: 1.1–2.3, respectively). However, the role of religion seemed to have no impact on condom use. Being of Protestant faith interacted with gender: among those who had debuted sexually, Protestant female students were more likely to have had three or more lifetime partners; the opposite was true for Protestant male students.

**Conclusion:**

Religion emerged as an important determinant of sexual behavior among Ugandan university students. Our findings correlate with the increasing number of conservative religious injunctions against premarital sex directed at young people in many countries with a high burden. of HIV/AIDS. Such influence of religion must be taken into account in order to gain a deeper understanding of the forces that shape sexual behavior in Uganda.

## Introduction

The early phase of the HIV/AIDS pandemic struck Uganda with great severity. In 1995 the estimated prevalence of HIV infection in the adult population was 15% [1]. However, the response in Uganda was quicker and more effective than in most similar countries [2], [3]. An unusual openness in acknowledging sexual behavior as the root of the epidemic has been credited with the broad mobilization of the voluntary sector, especially through the activities of faith-based organizations [4], [5]. The forthright discussion of sexuality did not seem to prevent religious institutions from supporting the preventive efforts, which were primarily based on the so-called ABC strategy: Abstinence from sex before marriage. Being faithful to one's partner, and using Condoms [6].

Such efforts reduced the prevalence of HIV/AIDS in the adult population to about 5% [7]. Increasing the availability of effective treatment also slowed the further spread of the virus. However, in order to sustain this initial success, the target group for HIV/AIDS prevention was logically the young population, i.e., youth from the onset of puberty up to the age of marriage [7], [8]. This group represents the most sexually active part of the population and as generally agreed, a sector highly sensitive to changes in behaviors—particularly sexual ones. Young people are also relatively easy to reach for preventive interventions via the educational institutions where they spend many years of their lives. Accordingly, since schools are the most likely arena for initiating sexual contacts, it is important to determine the most important factors that shape sexual behaviors in such settings. Previous studies have focused primarily on sociodemographic factors, including rapid urbanization, a growing middle class, and a tendency toward more active religious conservatism or fundamentalism [9]. The latter is of particular interest, since some religious organizations have increasingly challenged the ABC strategy, especially the condom component [10]. University populations, which largely represent the expanding middle class and have been the scene of growing activity by religious movements, have recently been investigated from this point of view [9].

In a global perspective, there are several studies of the relation between sexual behaviors and the financial and educational level of the household, and the influence of urban or rural setting elements [11], [12]. However, fewer studies have been done on religion and sexual behavior among young people in sub-Saharan Africa [9], [13], [14].

The importance of socioeconomic factors was demonstrated in a study on the estimated median age at first sex (AFS) among men and women born from the 1950 s through the 1980 s in Uganda. The findings showed an increase of AFS among women born after 1970, but not men. However, since results varied in comparing the neighboring districts of Masaka and Rakai, the authors suggest this might be explained by socioeconomic differences between those areas [15].

A cross-sectional study carried out among young people in Zimbabwe found religion to be a protective factor in sexual abstinence [14]. Similar results were shown in a population-based study involving young people between the ages of 15 to 24 in Côte d'Ivoire [16]. By contrast, an investigation of university students in Nigeria did not show any association between religion and sexual behavior [13]. At Makerere University in Uganda, more than half of twenty-five students interviewed who were members of the Kampala Pentecostal Church had engaged in sexual activity after being “born-again” [9]. This indicates the likelihood that the impact of religion on sexual behavior among young individuals is strongly dependent on other contextual factors and thus needs to be assessed separately for settings with similar characteristics, i.e., by country or by region.

Our knowledge of whether urbanization and a growing middle class, together with an increasing conservative religious movement, has changed the ABC pattern among university students in sub-Saharan Africa in general, or in Uganda in particular, is uncertain and even contradictory. Therefore, we lack the information to assess the implications these societal changes may have on the HIV/AIDS situation in this group and, consequently, on the prerequisites for successful intervention. Accordingly, the aim of this study was to investigate the relationship between sociodemographic and religious factors and their impact on sexual behavior among university students in Uganda.

## Methods

### Ethics statement

The research project was approved by the Institutional Ethical Review Committee at MUST.

### Population and setting

The study was performed at Mbarara University of Science and Technology (MUST), a public university in the town of Mbarara in southwestern Uganda. Founded in 1988, the school emphasizes community involvement in its teaching, fieldwork, and research. Our sample was drawn from the university's three faculties: medicine, science, and development studies, which at the time had a combined enrolment of 1220 undergraduate students, most of whom came from the surrounding area.

Data was collected by means of an 11 page self-administered instrument consisting of 132 questions. The development of the questionnaire was based on valid instruments used in other studies of a similar nature, and on the outcomes of focus group discussions with youth, including students in Mbarara district [17], [18], [19], [20], [21]. The questionnaire was developed in close collaboration with representatives of the students and was pre-tested by ten students. The formulation and interpretation of the more private and sensitive questions were also discussed with the student representatives. The questionnaires were distributed in lecture halls to all undergraduate students at MUST. Prior to the distribution, students were orally informed about the purpose of the questionnaires and were given instructions for filling them out. The completed questionnaires were to be turned in anonymously. Written informed consent was obtained for each participant. While the questionnaires were being filled out, the research staff ensured quiet and privacy. A total of 980 students responded to the questionnaire, representing 80% of all undergraduate students registered at the university.

The questionnaire assessed lifestyle factors (such as alcohol consumption, drug use, and smoking habits); relationships, love, and sexuality; social relations, participation, and trust (social capital); self-rated health, including mental health; and social and demographic factors (such as area of origin, socioeconomic status [SES], role of religion, and religious affiliation).

### Definition of variables

#### Background variables

Area of origin was dichotomized into “rural” and “urban/peri-urban or small town”.

The educational level of the head of household during childhood was dichotomized, so that “did not finish primary school” and “completed primary school” were coded as “low”, and “completed secondary school” and any education above that was classified as “high”.

The primary family religion during childhood was reported by selecting one of the following alternatives: “Protestant”, “Catholic”, “Moslem”, “Pentecostal”, “Seventh-day Adventist”, “Orthodox”, and “other”. In the final analysis only individuals reporting “Protestant” or “Catholic” denomination were used, since they make up the two major religions in our sample.

The role of religion in the family when growing up was dichotomized, so that “religion played a big role” and “religion was relatively important” were coded as “big role”; and “religion was not so important” and “religion was not important at all” were coded as “not big role”.

Faculty of study was reported and categorized as “medicine”, “development studies”, or “science”.

Two age groups were established for our analysis: “younger” (≤23 years old), and “older” (>23 years old).

Self-related health was defined on the basis of responses to the question: “How do you classify your current health in general?” There were five alternative answers: “very bad”, “bad”, “fair”, “good”, and “very good”. In the following analyses, the variable was dichotomized into less good (the first two alternatives) and good (the remaining three alternatives).

#### Sexual behavior variables: dependent variables

The variable for having previously had sexual intercourse was defined as “yes” or “no”, based on responses to the question: “Have you ever had sexual intercourse?”

Age at sexual debut was dichotomized, so that having sexual intercourse for the first time before age 19 was coded as “low”, and at or above age 19 as “high”.

Number of lifetime sexual partners was defined by responses to the question: “How many sexual partners have you had altogether?” The variable was then dichotomized, so that ≥3 was coded as “high”, and <3 was coded as “low”.

Condom use with new partner was measured by responses to the question: “How often do you use a condom with a new sexual partner?” Respondents could answer by choosing any of five alternatives: “always”, “often”, “sometimes”, or “never”. This was then dichotomized by keeping the first alternative as “always” and the latter three as “not always”.

Condom use on latest occasion of sexual intercourse was assessed through responses to the question: “Did you use any method for avoiding STDs on your latest occasion of sexual intercourse?” There were three alternative answers: “no”, “yes, condom”, and “yes, other”. The variable was then dichotomized, so that “no” and “yes, other” were coded as “inconsistent”, and “yes, condom” was coded as “consistent”.

### Statistical methods

Sample size was given since we assessed all the students at the university, but a formal check revealed that in most analyses a 75% increase of risk could be ascertained at 80% probability. This did not exclude the risk of not being able to detect some true effects of moderate size.

The statistical analyses were done with SPSS Version 16.0. Logistic regressions were performed to calculate the crude odds ratios (OR) and 95% confidence intervals (CI) for the effect on age at sexual debut, having previously had sex, number of lifetime sexual partners, and consistent condom use. Differences between men and women in the prevalence of the variables used were calculated by means of Chi-square values upon which the *p*-values shown in Table 1 were based. Only cases where information was available on all variables in a particular instance were analyzed.

**Table 1:** Prevalence of socio–demographic factors, self–rated health, and sexual behavior factors.

	All		Male		Female		x^2^
	n	%	n	%	n	%	*p*
*Sex*							
Male	633	64.6					
Female	347	35.4					
*Age*							
Younger ≤23	628	65.6	378	60.6	250	75.1	<0.001
Older ≥24	329	34.4	246	39.4	83	24.9	
Missing	(23)		(9)		(14)		
*Area of origin*							
Rural	424	43.7	318	50.6	106	31.0	<0.001
Urban/peri–urban	546	56.3	310	49.4	236	69.0	
Missing	(10)		(5)		(5)		
*Educational level of head of household*							
≤Primary	235	25.5	186	31.0	49	15.2	<0.001
>Primary school	688	74.5	414	69.0	274	84.8	
Missing	(57)		(33)		(24)		
*Religious affiliation*							
Protestant	415	42.8	273	43.5	142	41.4	0.44
Catholic	379	39.1	248	39.6	131	38.2	
Moslem	86	8.9	56	8.9	30	8.7	
Pentecostal	45	4.6	24	3.8	21	6.1	
Seventh Day Adventist	22	2.3	15	2.4	7	2.0	
Orthodox	8	0.8	4	0.6	4	1.2	
Other	15	1.5	7	1.1	8	2.3	
Missing	(10)		(6)		(4)		
*Importance of religion*							
Big role	542	55.9	337	53.8	205	59.8	0.08
Not big role	427	44.1	289	46.2	138	40.2	
Missing	(11)		(7)		(4)		
*Self–rated health*							
Good	730	85.1	476	85.9	254	83.6	0.37
Less good	128	14.9	78	14.1	50	16.4	
Missing	(122)		(79)		(43)		
*Previously had sex*							
Yes	532	59.0	376	62.9	156	51.3	0.001
No	370	41.0	222	37.1	148	48.7	
Missing	(78)		(35)		(43)		
*Age at sexual debut* 1		51.2					
≤18 = low	262	51.2	199	55.0	63	42.0	0.01
>18 = high	250	48.8	163	45.0	87	58.0	
Missing	(20)		(14)		(6)		
*Number of lifetime sexual partners* 1							
1−2 = low	293	61.0	180	54.1	113	76.9	<0.001
≥3 = high	187	39.0	153	45.9	34	23.1	
Missing	(52)		(43)		(9)		
*Condom use with a new partner* 1							
Always	324	66.7	235	68.5	89	62.2	0.21
Not always	162	33.3	108	31.5	54	37.8	
Missing	(46)		(33)		(13)		
*Used a condom on latest occasion of sexual intercourse* 1							
Consistent	424	82.7	306	85.2	118	76.6	0.02
Inconsistent	89	17.3	53	14.8	36	23.4	
Missing	(19)		(17)		(2)		

1 Only analysed among individuals who had had sexual intercourse.

In order to estimate the mean age of sexual debut, we performed a survival analysis with our data by making age at first sex right-censored since some people had not yet had sex.

Multivariate logistic regression stratified by sex was used to investigate the association between role of religion, religious affiliation, and sexual behavior. The effect of these variables on behavioral factors (i.e., age at sexual debut, having previously had sex, number of lifetime sexual partners, and condom use) was adjusted for age and area of origin. OR and 95% CI were used as measures of association.

An additional analytical step was taken to determine whether there was effect modification between the variables chosen. Effect modification is present when a certain factor has a greater impact on an outcome in the presence or absence of a third variable (e.g., one exposure affects the risk of disease in men, but not in women).

The test for effect modification was made as a test for “more than additivity”, according to Rothman [22]. It is technically performed in the following way: stratified analyses are made for two of the involved exposure variables (e.g., gender and importance of religion) in order to detect possible effect measure modification of the odds ratios. Four dummy variables were created with the values a) female + high importance of religion, b) male + high importance of religion, c) female + low importance of religion, and d) male + low importance of religion. Possible synergy effects concerning gender and importance of religion on the outcome variables (sexual behaviors) were assessed by using the algorithm:

SI = OR(AB)−1/(OR(Ab)−1)+(OR(aB)−1) OR = odds ratio; AB = exposed to both factors; Ab = exposed to one of the factors, and aB = exposed to the other factor. In the absence of effect modification, SI = 1, i.e., there is a pure additive effect when both exposures are present. In case of synergy, SI is greater than 1 (i.e., “more than additivity”); in case of antagonism, SI is less than 1.

## Results

A total of 980 students responded to the questionnaire, representing 80% of all (n = 1220) registered students. Thirty-five percent (n = 347) of the respondents were female and 65% (n = 633) were male.

Table 1 shows the distribution of sociodemographic factors and self-rated health, as well as the outcome variables: having previously had sexual intercourse, age at sexual debut, number of lifetime sexual partners, consistent condom use, and use of a condom on latest occasion of sexual intercourse. The majority of the male and female students (69.0% and 84.8%, respectively) grew up with a head of household who had high educational level (secondary school or above). In all, 54.8% of the males and 59.8% of the females reported that religion played a big role in their family of origin. Significantly more males than females reported having previously had sexual intercourse (62.9% vs. 51.3%, respectively). About half of the students had made their sexual debut by age 18. In fact, a survival analysis was performed to estimate the mean age of sexual debut, which was 17.9 years (95% CI: 17.5–18.2) in the sample (data not shown).

In the group that stated they had previously had sex, 45.9% of the males and 23.1% of the females reported having had three or more sexual partners. More females (37.8%) than males (31.5%) stated that they do not always use a condom with a new partner.

Tables 2, 3, 4, and 5 provide an analysis of the associations in the population studied between sociodemographic factors, on the one hand, and self-rated health and sexual behavior, on the other. Rural origin had a statistically significant association with previously having had sexual intercourse among male students (OR 1.5, 95% CI 1.1–2.1). Protestant religious affiliation was negatively associated with having previously had sexual intercourse among female students (OR 0.5, 95% CI 0.3–0.8), compared to Catholic female students. Protestant male students had a statistically lower risk for having had a high number of lifetime sexual partners (OR 0.6, 95% CI 0.4–0.96). Among male students, a larger proportion in the group stating that religion did not play a big role in their family had an increased risk of early sexual debut (OR 1.7, 95% CI 1.1–2.6), and female students in the same group had a greater risk of having had a high number of lifetime sexual partners (OR 2.2, 95% CI 1.01–4.8). No statistically significant association was found between self-rated health and previously had sex, age of sexual debut, number of lifetime partners, and condom use, and no association was found between role of religion and condom use.

**Table 2:** Association (OR, 95% CI) between socio–demographic factors, self–rated health, and *previously had sex*.

Sociodemographic background factors	Previously had sexAll	Male	–Female
	OR (95% CI)	OR (95% CI)	OR (95% CI)
*Sex*			
Male	1 (ref)		
Female	0.6 (0.5–0.8)		
*Age*			
Younger	1 (ref)	1 (ref)	1 (ref)
Older	2.1 (1.6–2.8)	1.9 (1.3–2.7)	2.2 (1.3–3.8)
*Area of origin*			
Urban/peri–urban small town	1 (ref)	1 (ref)	1 (ref)
Rural	1.5 (1.1–1.9)	1.5 (1.1–2.1)	1.2 (0.7–1.9)
*Educational level of head of household*			
High	1 (ref)	1 (ref)	1 (ref)
Low	1.0 (0.8–1.4)	0.9 (0.6–1.3)	1.1 (0.6–2.2)
*Religious affiliation*			
Catholic	1 (ref)	1 (ref)	1 (ref)
Protestant	0.9 (0.7–1.3)	1.2 (0.8–1.8)	0.5 (0.3–0.8)
*Role of religion*			
Big	1 (ref)	1 (ref)	1 (ref)
Not Big	1.2 (0.96–1.6)	1.4 (0.98–1.9)	1.0 (0.6–1.5)
*Faculty affiliation*			
Medicine	1 (ref)	1 (ref)	1 (ref)
Other	1.2 (0.9–1.6)	1.1 (0.8–1.6)	1.4 (0.9–2.2)
*Self–rated health*			
Good	1 (ref)	1 (ref)	1 (ref)
Less good	1.0 (0.7–1.5)	1.1 (0.6–1.8)	1.0 (0.5–1.8)

**Table 3:** Association (OR, 95% CI) between sociodemographic factors, self–rated health, and *low age at sexual debut*.

Sociodemographic background factors	Low age of sexual debutAll	Male	Female
	OR (95% CI)	OR (95% CI)	OR (95% CI)
*Sex*			
Male	1 (ref)		
Female	0.6 (0.4–0.9)		
*Age*			
Younger	1 (ref)	1 (ref)	1 (ref)
Older	0.7 (0.5–0.99)	0.6 (0.4–0.9)	0.8 (0.4–1.7)
*Area of origin*			
Urban/peri–urban small town	1 (ref)	1 (ref)	1 (ref)
Rural	1.0 (0.7–1.4)	1.0 (0.6–1.5)	0.6 (0.3–1.3)
*Educational level of head of household*			
High	1 (ref)	1 (ref)	1 (ref)
Low	1.1 (0.7–1.6)	0.9 (0.6–1.5)	1.3 (0.5–3.2)
*Religious affiliation*			
Catholic	1 (ref)	1 (ref)	1 (ref)
Protestant	0.9 (0.6–1.4)	0.9 (0.6–1.5)	0.7 (0.4–1.6)
*Role of religion*			
Big	1 (ref)	1 (ref)	1 (ref)
Not big	1.7 (1.2–2.4)	1.7 (1.1–2.6)	1.6 (0.8–3.1)
*Faculty affiliation*			
Medicine	1 (ref)	1 (ref)	1 (ref)
Other	0.8 (0.6–1.2)	0.8 (0.5–1.2)	1.1 (0.5–2.1)
*Self–rated health*			
Good	1 (ref)	1 (ref)	1 (ref)
Less good	0.9 (0.6–1.6)	1.0 (0.5–1.9)	0.9 (0.3–2.3)

**Table 4:** Association (OR, 95% CI) between socio–demographic factors, self–rated health, and *high number of lifetime sexual partners*.

Sociodemographic background factors	High number of life–time sexual partnersAll	Male	Female
	OR (95% CI)	OR (95% CI)	OR (95% CI)
*Sex*			
Male	1 (ref)		
Female	0.4 (0.2–0.6)		
*Age*			
Younger	1 (ref)	1 (ref)	1 (ref)
Older	1.3 (0.9–1.9)	1.1 (0.7–1.7)	1.8 (0.8–4.1)
*Area of origin*			
Urban/peri–urban small town	1 (ref)	1 (ref)	1 (ref)
Rural	1.1 (0.8–1.6)	0.9 (0.6–1.3)	0.9 (0.4–2.1)
*Educational level of head of household*			
High	1 (ref)	1 (ref)	1 (ref)
Low	1.4 (0.9–2.2)	1.3 (0.8–2.0)	1.0 (0.3–2.9)
*Religious affiliation*			
Catholic	1 (ref)	1 (ref)	1 (ref)
Protestant	0.9 (0.6–1.3)	0.6 (0.4–0.9)	2.0 (0.8–4.9)
*Role of religion*			
Big	1 (ref)	1 (ref)	1 (ref)
Not big	1.6 (1.1–2.3)	1.3 (0.9–2.0)	2.2 (1.01–4.8)
*Faculty affiliation*			
Medicine	1 (ref)	1 (ref)	1 (ref)
Other	0.8 (0.6–1.2)	0.9 (0.5–1.3)	0.8 (0.4–1.8)
*Self–rated health*			
Good	1 (ref)	1 (ref)	1 (ref)
Less good	1.1 (0.6–1.8)	1.2 (0.6–2.3)	1.0 (0.3–3.0)

**Table 5:** Association (OR, 95% CI) between sociodemographic factors, self–rated health, and *not always condom use with a new partner*.

Sociodemographic background factors	Not always condom use with a new partnerAll	Male	Female
	OR (95% CI)	OR (95% CI)	OR (95% CI)
*Sex*			
Male	1 (ref)		
Female	1.3 (0.9–2.0)		
*Age*			
Younger	1 (ref)	1 (ref)	1 (ref)
Older	1.0 (0.7–1.5)	1.1 (0.7–1.7)	1.0 (0.5–2.0)
*Area of origin*			
Urban/peri–urban small town	1 (ref)	1 (ref)	1 (ref)
Rural	1.1 (0.8–1.6)	1.1 (0.7–1.7)	1.1 (0.6–2.3)
*Educational level of head of household*			
High	1 (ref)	1 (ref)	1 (ref)
Low	1.1 (0.7–1.8)	1.1 (0.7–1.9)	1.5 (0.6–4.1)
*Religious affiliation*			
Catholic	1 (ref)	1 (ref)	1 (ref)
Protestant	0.9 (0.6–1.3)	1.1 (0.7–1.7)	0.6 (0.3–1.3)
*Role of religion*			
Big	1 (ref)	1 (ref)	1 (ref)
Not big	0.9 (0.6–1.3)	1.0 (0.6–1.5)	1.0 (0.5–1.7)
*Faculty affiliation*			
Medicine	1 (ref)	1 (ref)	1 (ref)
Other	1.1 (0.7–1.6)	1.0 (0.6–1.6)	1.3 (0.6–2.6)
*Self–rated health*			
Good	1 (ref)	1 (ref)	1 (ref)
Less good	1.6 (0.9–2.7)	1.7 (0.9–3.2)	1.3 (0.5–3.3)

Based on these findings, two variables (role of religion and religious affiliation) were chosen as the main determinants of sexual behaviors for further investigation. We adjusted for potential confounding due to age and rural origin by employing multi-variate logistic regression.

Tables 6 and 7 presents the adjusted OR with 95% CI for associations between the determinants mentioned and religious affiliation vis-à-vis the dependent variables (i.e., the sexual behaviors studied) stratified by sex. Two models were used: the first one adjusted for age, and the second for age and area of origin. As seen in the tables, the associations between role of religion and having previously had sex, age of sexual debut, and number of lifetime sexual partners only changed marginally, even after adjusting for age and rural origin.

**Table 6:** Association (OR 95% CI) between role of religion and sexual behavior.

Sexual behavior factor	Model 1		Model 2	
	(adjusted for age)		(adjusted for age and area of origin)	
	Female (n = 304)	Male (n = 598)	Female (n = 299)	Male (n = 593)
*Previously had sex*				
Not big role of religion	1.0 (0.6–1.7)	1.4 (0.98–2.0)	1.0 (0.6–1.6)	1.4 (0.98–2.0)
Older	2.2 (1.3–3.8)	1.4 (0.98–1.9)	2.3 (1.3–4.0)	1.9 (1.3–2.7)
Rural			1.1 (0.6–1.8)	1.4 (0.99–2.0)
*Low age of sexual debut*				
Not big role of religion	1.7 (0.9–3.3)	1.5 (0.99–2.3)	1.7 (0.9–3.4)	1.5 (1.01–2.4)
Older	0.9 (0.4–1.8)	0.6 (0.4–0.9)	0.9 (0.4–1.8)	0.6 (0.4–0.9)
Rural			0.6 (0.3–1.2)	1.0 (0.6–1.5)
*High number of lifetime sexual partners*				
Not big role of religion	2.8 (1.2–6.4)	1.3 (0.8–2.0)	2.8 (1.2–6.5)	1.3 (0.9–2.1)
Older	2.3 (0.97–5.3)	1.1 (0.7–1.8)	2.3 (0.97–5.3)	1.1 (0.7–1.8)
Rural			0.7 (0.3–1.8)	0.9 (0.6–1.3)
*Not always condom use with a new partner*				
Not big role of religion	0.9 (0.5–1.9)	1.0 (0.7–1.6)	0.9 (0.5–1.8)	1.1 (0.7–1.7)
Older	1.0 (0.5–2.0)	1.1 (0.7–1.7)	1.0 (0.5–2.0)	1.0 (0.7–1.6)
Rural			1.1 (0.5–2.3)	1.1 (0.7–1.8)

**Table 7:** Association (OR 95% CI) between religious affiliation and sexual behavior.

Sexual behavior factor	Model 1		Model 2	
	(adjusted for age)		(adjusted for age and area of origin)	
	Female	Male	Female	Male
*Previously had sex*				
Protestant	0.5 (0.3–0.9)	1.2 (0.8–1.8)	0.5 (0.3–0.9)	1.2 (0.8–1.7)
Older	2.5 (0.3–0.9)	2.2 (1.5–3.3)	2.5 (1.3–4.8)	2.1 (1.4–3.2)
Rural			1.2 (0.7–2.2)	1.5 (1.01–2.2)
*Low age of sexual debut*				
Protestant	0.7 (0.3–1.5)	0.9 (0.6–1.5)	0.8 (0.4–1.8)	0.9 (0.6–1.5)
Older	0.5 (0.2–1.2)	0.6 (0.4–0.97)	0.5 (0.2–1.2)	0.6 (0.4–0.97)
Rural			0.5 (0.2–1.1)	1.0 (0.6–1.5)
*High number of lifetime sexual partners*				
Protestant	2.0 (0.8–5.0)	0.6 (0.4–0.97)	2.1 (0.9–5.4)	0.6 (0.4–0.99)
Older	1.6 (0.6–4.1)	0.9 (0.6–1.5)	1.6 (0.6–4.2)	1.0 (0.6–1.6)
Rural			0.7 (0.3–2.1)	0.8 (0.5–1.3)
*Not always condom use with a new partner*				
Protestant	0.6 (0.3–1.3)	1.1 (0.7–1.8)	0.6 (0.3–1.4)	1.1 (0.7–1.8)
Older	0.7 (0.3–1.7)	1.0 (0.6–1.6)	0.7 (0.3–1.7)	1.0 (0.6–1.6)
Rural			0.7 (0.3–1.7)	1.4 (0.9–2.4)

Since the aim of the study was to analyze the pattern of associations between sociodemographic and religious factors that correlate with previously had sex, age of sexual debut, number of sexual partners, and condom use, we next, in Tables 8, 9, 10, and 11, analyzed possible effect modification between gender and role of religion, as well as type of religion in conjunction with the sexual behavior factors studied.

**Table 8:** Analysis of effect modification between sex and role of religion/religious affiliation regarding *previously had sex* in a sample of Ugandan university students (n = 980), presented as adjusted OR with 95% CI.

Sex and role of religion/religious affiliation	Previously had sexAll	
	n (%)	OR (95% CI)^1^
*Sex/role of religion*		
Female/Big role	205 (21.2)	1 (ref)
Female/Not big role	138 (14.2)	1.0 (0.6–1.7)
Male/Big role	337 (34.8)	1.3 (0.9–1.9)
Male/Not big role	289 (29.5)	1.8 (1.2–2.7)
(Missing)	(11)	
Total	980	
*Sex/religious affiliation*		
Female/Protestant	142 (14.6)	1 (ref)
Female/Other religion	201 (20.5)	1.8 (1.1–2.9)
Male/Protestant	273 (27.9)	2.5 (1.6–4.0)
Male/Other religion	354 (36.1)	2.0 (1.5–2.8)
(Missing)	(10)	
Total	980	

1 Adjusted for age.

**Table 9:** Analysis of effect modification between sex and role of religion/religious affiliation regarding *low age of sex debut* in a sample of Ugandan university students (n = 980), presented as adjusted OR with 95% CI.

Sex and role of religion/religious affiliation	Low age of sex debutAll	
	n (%)	OR (95% CI)^1^
*Sex/role of religion*		
Female/Big role	205 (21.2)	1 (ref)
Female/Not big role	138 (14.2)	1.6 (0.8–3.2)
Male/Big role	337 (34.8)	1.9 (1.1–3.2)
Male/Not big role	289 (29.5)	2.9 (1.7–5.0)
(Missing)	(11)	
Total	980	
*Sex/religious affiliation*		
Female/Protestant	142 (14.6)	1 (ref)
Female/Other religion	201 (20.5)	1.4 (0.7–2.9)
Male/Protestant	273 (27.9)	2.3 (1.1–4.5)
Male/Other religion	354 (36.1)	2.5 (1.3–5.0)
(Missing)	(10)	
Total	980	

1 Adjusted for age.

**Table 10:** Analysis of effect modification between sex and role of religion/religious affiliation regarding *high number of lifetime sexual partners* in a sample of Ugandan university students (n = 980), presented as adjusted OR with 95% CI.

Sex and role of religion/religious affiliation	High number of lifetime sexual partnersAll	
	n (%)	OR (95% CI)^1^
*Sex/role of religion*		
Female/Big role	205 (21.2)	1 (ref)
Female/Not big role	138 (14.2)	2.5 (1.1–5.7)
Male/Big role	337 (34.8)	3.8 (2.0–7.2)
Male/Not big role	289 (29.5)	5.1 (2.7–9.8)
(Missing)	(11)	
Total	980	
*Sex/religious affiliation*		
Female/Protestant	142 (14.6)	1 (ref)
Female/Other religion	201 (20.5)	0.6 (0.3–1.3)
Male/Protestant	273 (27.9)	1.6 (0.8–3.3)
Male/Other religion	354 (36.1)	2.5 (1.2–5.0)
(Missing)	(10)	
Total	980	

1 Adjusted for age.

**Table 11:** Analysis of effect modification between sex and role of religion/religious affiliation regarding, *not always condom use with a new partner* in a sample of Ugandan university students (n = 980), presented as adjusted OR with 95% CI.

Sex and role of religion/religious affiliation	Not always condom use with a new partnerAll	
	n (%)	OR (95% CI)^1^
*Sex/role of religion*		
Female/Big role	205 (21.2)	1 (ref)
Female/Not big role	138 (14.2)	1.0 (0.5–2.1)
Male/Big role	337 (34.8)	0.8 (0.5–1.4)
Male/Not big role	289 (29.5)	0.8 (0.5–1.4)
(Missing)	(11)	
Total	980	
*Sex/religious affiliation*		
Female/Protestant	142 (14.6)	1 (ref)
Female/Other religion	201 (20.5)	1.3 (0.6–2.8)
Male/Protestant	273 (27.9)	1.1 (0.5–4.1)
Male/Other religion	354 (36.1)	0.8 (0.4–1.7)
(Missing)	10	
Total	980	

1 Adjusted for age.

Effect modification between gender and role of religion was demonstrated regarding having previously had sex: males reporting that religion did not play a big role had a higher probability of having previously had sex than males for whom religion was rated as important, a pattern not visible found among females. Effect modification between gender and type of religion, i.e. Protestant vs. Catholic, was also demonstrated with regard to previously having had sex. We found that type of religion had opposite outcomes among men and women. In Protestant male students, the likelihood of having previously had sex increased, while it decreased among women of the same denomination.

Furthermore, effect modification between gender and religious affiliation was demonstrated regarding lifetime sexual partners. Being a Protestant had opposite effects for men and women. No effect modification between gender and role of religion appeared with regard to condom use, since none of the combinations of the exposure variables mentioned appeared to affect the outcome. The same was largely true concerning effect modification between gender and religious affiliation and condom use. However, Protestant male students tended to be associated with a higher risk of not using a condom, compared with female Protestant students.

## Discussion

Our study demonstrated a statistically significant correlation between two sets of factors: importance of religion and religious denomination in relation to sexual debut and number of lifetime sexual partners.

Gender tended to modify the effect of role of religion. Responses stating that religion played a big role in one's family were associated with previously having had sex among males, but not among females. Gender also seemed to modify the effect of religious affiliation. Being of Protestant faith was associated with higher risk for previously having had sex among males, but with lower risk among females. Protestant male students showed a lower probability of having many lifetime sexual partners compared with Catholic male students, while the opposite seemed to be the case among Protestant female students.

The results must be interpreted in relation to its rather exclusive target group, and the generalization of our findings to all individuals of the same age should be made with care. The majority of those in the target group have most probably grown up in a more protected environment than others in their age group. More than two-thirds of the students were raised in an urban or suburban area by a head of household who had a high educational level. This is not representative of young people in Uganda in general, only 14% of whom live in urban settings, according to the 2002 National Census. Of the population ages 20 to 24 years old, 8.9% was enrolled in post-secondary (higher) education [23]. Moreover, the target group consists of a mainly unmarried population: there are very few married students (if any) registered in the university [24].

This study was performed by means of a cross-sectional design, which may present difficulties in ascertaining the direction of causality between the variables analyzed. However, the sociodemographic variables were all unlikely to be affected by the sexual behaviors that constituted the outcome variable, since the circumstances involved (e.g., a family's religious affiliation or the educational level of the head of household) chronologically preceded the outcome.

As noted, more than 80% of all students at the university completed the questionnaire. Most of the remaining 20% could not be contacted by the research team because they were not on campus for various reasons. The “true” rate of non-responders was, therefore, less than 20% (and probably below 5%). Although we did not have access to information on how many students were off-campus, we know that 66% of the total number of students were males and 34% were females. The distribution of males and females in the remaining 20% (240 students) was 72% (n = 172) for males and 28% (n = 68) for females. Under these circumstances, it seems unlikely that systematic factors should have caused any selection bias of importance for the results. Internal missing was on the order of 5% to 10% regarding questions concerning sexual behavior. This could lead one to infer a moderate selection bias in an unknown direction, although the likelihood that this would have dramatically biased the risk is low.

The issue of misclassification should also be considered. We could not rule out the possibility that some of the respondents underreported sexual behavior that would be viewed as socially undesirable. If this were done in association with the background factors (e.g., the role of religion), it could represent dependent misclassification and, in such a case, would most likely exaggerate the findings. Thus, an alternative explanation to the suggested effect modification by gender would be that there may have been differential reporting of sexual behavior by the men and women in our sample, i.e., stronger moral rules might have made women underreport “unacceptable” sexual behavior to a greater extent than men did. However, upon scrutinizing our findings, this does not seem to be a very systematic pattern, although it cannot be ruled out. Moreover, respondents were guaranteed anonymity, and when the outcome of the questionnaire was discussed with student representatives, they stated that this guarantee was taken seriously. In their judgment the results gave a realistic picture of the true circumstances.

Among possible confounders the most obvious one was age. All final risk estimates were, therefore, controlled for this factor. Since being of rural origin was also a predictor of sexual behavior, this variable was included as a potential confounder in the final multivariate model. However, one might object that this could result in a measure of overadjustment, since the variable could be a determinant of religious engagement, thus representing a single possible pathway. Because the other sociodemographic factors did not appear significantly related to the outcome variables, they were not considered potential confounders. In summary, even though the most plausible confounders were controlled for, this did not change the risk estimates.

Age was used as a dichotomous variable in the analyses. We made supplementary analyses which very clearly showed that age and sexual debut/number of sexual partners were not related in a linear way as a simple function of time (i.e., age). Rather, there is evidence for both a period and cohort effect. Therefore, we found it justifiable to group all individuals into two age groups. (In fact, we also performed all the analyses with a continuous age variable, but the results changed only marginally.)

The results of our study indicated a relatively low level of risky sexual behavior among the majority of students. Religious influences and denominational networks might partly explain this.

Our findings are in agreement with those of previous research performed mainly in settings outside Africa, where it was concluded that religious engagement was a protective factor for risky sexual behavior [25], [26], [27]. Such research has primarily studied the associations between religious engagement and religious affiliation in relation to sexual behavior in general. In a study performed in the US by Kindler, religion was categorized in four dimensions: personal devotion (a sense of personal connection to a god), personal conservatism (rigid or literal adherence to the creed of a religious denomination), institutional conservatism (fundamentalism of a religious denomination), and participation in a religious community [28]. A study by Miller & Gur [27] on religiosity and sexual responsibility focused on the associations between the above mentioned four religious dimensions and sexual behavior. The results showed that religious dimensions were variously self-identified with sexual behavior. Three of the four dimensions (personal devotion, frequent attendance, and institutional conservatism) were linked to a lower number of sexual partners in the previous year; however, young women who associated with personal conservatism tended to have a higher number of partners. However, no association with any of the four religious dimensions was found regarding abstinence.

Religious affiliation in Uganda is a lifetime commitment whose practices and rituals becomes part of an individual's daily experience. If one is born Catholic, she or he will be baptized Catholic, will go to Catholic schools, is expected to marry someone from a Catholic family, and will vote for Catholic-leaning politicians. Since it is uncommon to change one's religious affiliation (especially before marriage), it is highly likely that a person's *present* religious practices are directly related to the religion with which one grew up. As a result, hence an association may be drawn between past and present religious affiliation and current sexual behavior.

Although the Protestant Church only represents the second largest religion in Uganda, the western region of the country in which Mbarara University is located is predominately Protestant. That denomination exerts a strong political and social influence on many aspects of public and private life in Uganda. Religion among conservative Protestants in Uganda has increasingly become an exclusive “club” in which membership is obtained through strict Christian indoctrination. For example, students at Makerere University in Kampala, the capital of Uganda, are invited to join both a Christian Union and a care group (a spiritual mentorship team within the same church) when they enter the university. These two organizations distribute cards for students to sign pledging themselves to “abstinence until marriage”. Students are also invited to attend “virginity rallies” [29].

According to informants in Mbarara, “subgroups” or networks of conservative Protestant students at MUST stage similar activities and recruit new members during the first few days of each semester. The groups have the financial resources to mount attractive networking activities. In order for students to join, they are obligated to follow certain rules of conduct (e.g., it is not acceptable to be involved in any sexual relationship). Whoever does not abide by the rules is reported to the group leaders.

In our judgment, the Protestant group at MUST seems most akin to Kindler's category “participation in a religious community” cited above. Institutional conservatism may also be implied because of the character of the Protestant Church in Uganda.

Our survey showed a similar pattern of behavior among Protestant female students regarding condom use as identified in Miller & Gur's study as their institutional conservative group, i.e., neither differed from other individuals. However, most sexual relationships involving a Protestant female student in Uganda take place in secrecy, according to informants, which differs from the findings reported by Miller & Gur. Moreover, the impact of religion may depend on the specific cultural context. A Ugandan Protestant young woman involved in a sexual relationship may be more likely to use condoms for fear of pregnancy, which would expose her clandestine behavior and subject her to condemnation by her family and coreligionists.

Our findings also showed a lower risk for having had sexual intercourse among Protestant female students. In Uganda, this might be because Protestant girls are brought up in a closed system of rigid institutionalized choices, less exposed to the “outside” world, and thus less likely to engage in early sexual activities. In previous research conservative Protestantism has been described as both a cultural phenomenon and a religious subculture that protects or insulates the individual from external secularizing trends [30], [31].

We found that importance of religion and religious affiliation was associated with abstinence. In a recent study Muslim men were less likely to practice abstinence than Protestants, Pentecostals, and Catholic male youth [32]. The default settings for acceptable relationships are very complex. Churches in Uganda demand that members seek partners with the same religious affiliation. Moreover, church leaders must give their sanction before a marital union is concluded. In the meanwhile, the message continues to be abstinence and all church members are expected to adhere to this creed. As a result, a young person who, for whatever reason, is tempted to have sex will most likely have difficulty obtaining a condom from friends or family members, who will associate premarital sex with immorality and religious backsliding. In addition, in Uganda condoms are mostly sold over-the-counter in shops or pharmacies where one needs to ask the shop attendant for them “publicly”, making condoms hard to purchase in a clandestine manner. In effect, a religious young man buying a condom in the marketplace stigmatizes himself. Consequently, young people, especially males, end up having unprotected sex within secret relationships.

Not only is, premarital sex is unacceptable to religious individuals in Uganda: it is believed that becoming pregnant stigmatizes not only the individual, but the church as well. Thus, the demand that members practice abstinence before marriage is not debatable, regardless of whether an individual is planning to marry in the near future. According to interviews with 15 to 24-year-old young people in the Mityana and Mubende districts of Uganda, their society expects that a person abstain from sex before marriage, i.e., remain a virgin. Moreover, the prevailing norms do not support the use of condoms or any other method of contraception [33]. In a study conducted among young people in Kampala and in a Wakiso district village 18 km outside the city, the most ideal relationship was described as one between a young man and woman of the same age and religious affiliation. In addition, it was agreed that sexual activities should be postponed until after one's education is concluded and both parties reach at least the age of 18 [34].

The increasing impact of religion and religious affiliation is very obvious in Ugandan society, and impacts most young people's daily lives in Uganda. Religious beliefs and attitudes on sexual matters appear to have considerable influence on the sexual conduct of Ugandan young men and women. However, the causal pathway between religion and sexual behaviors among university students is likely to be complex. Interaction between such factors as social capital, social norms, and influence of peers may play a role. This was illustrated in a recent study in which students from a university in Uganda described themselves as being in the middle of an emerging clash of sexual ideologies [9].

The results of the same study showed that 11 out of 20 students who are members of the Pentecostal Church had been involved in sexual activities. This indicates that even a fundamentalist religious commitment does not rule out sexual activity among young people, despite the church proclaiming the opposite.

It is unclear if the control mechanisms of religion generate behavior modification through fear or through conviction. However, one may conjecture that fear of consequences plays a significant role in explaining why religious affiliation has had a more conservative effect on females. The gender imbalance rooted in the social culture and property relations in Uganda influences the power of decision making in sexual relationships. Uganda is predominately a masculine society in which men also control matters of sex [35], leaving women less empowered to make independent decisions in this regard. Gender roles in sexual relationships are reinforced by religious and social mores. As a result, women are often left with no choice but to bear the consequences of “inappropriate sexual behavior”.

### Conclusion

Religious factors appear to be important determinants of sexual behavior among Ugandan university students. Our findings should be viewed against the background of the increasing role of conservative religious injunctions against premarital sex among young people that is evident in many countries with a high burden of HIV/AIDS. Thus, the influence of religion must be taken into account in order to gain a deeper understanding of the forces that shape sexual behavior in Uganda. With such knowledge, one may design and implement more effective interventions to prevent the spread of sexually transmitted infections. However, the interplay between religion and gender is very complex and only some facets can be captured by means of quantitative methods. Additional light might, therefore, be shed on those issues by means of complementary studies using qualitative methods. Therefore, further research is needed to fully comprehend the mechanisms by which conservative religious beliefs and the social attitudes that result from them inadvertently promote risky sexual behavior.
